# Satellite-Based Analysis of Evapotranspiration and Water Balance in the Grassland Ecosystems of Dryland East Asia

**DOI:** 10.1371/journal.pone.0097295

**Published:** 2014-05-20

**Authors:** Jiangzhou Xia, Shunlin Liang, Jiquan Chen, Wenping Yuan, Shuguang Liu, Linghao Li, Wenwen Cai, Li Zhang, Yang Fu, Tianbao Zhao, Jinming Feng, Zhuguo Ma, Mingguo Ma, Shaomin Liu, Guangsheng Zhou, Jun Asanuma, Shiping Chen, Mingyuan Du, Gombo Davaa, Tomomichi Kato, Qiang Liu, Suhong Liu, Shenggong Li, Changliang Shao, Yanhong Tang, Xiang Zhao

**Affiliations:** 1 State Key Laboratory of Remote Sensing Science, Jointly Sponsored by Beijing Normal University and Institute of Remote Sensing and Digital Earth, Chinese Academic of Sciences, Beijing, China; 2 College of Global Change and Earth System Science, Beijing Normal University, Beijing, China; 3 Department of Geographical Sciences, University of Maryland, College Park, Maryland, United States of America; 4 International Center for Ecology, Meteorology and Environment (IceMe), School of Applied Meteorology, Nanjing University of Information Science and Technology, Nanjing, China; 5 State Key Laboratory of Earth Surface Processes and Resource Ecology, Beijing Normal University, Beijing, China; 6 State Engineering Laboratory of Southern Forestry Applied Ecology and Technology, Central South University of Forestry and Technology, Changsha, Hunan, China; 7 State Key Laboratory of Vegetation and Environmental Change, Institute of Botany, Chinese Academy of Sciences, Beijing, China; 8 Key Laboratory of Digital Earth Science, Institute of Remote Sensing and Digital Earth, Chinese Academy of Sciences, Beijing, China; 9 Key Laboratory of Regional Climate-Environment Research for Temperate East Asia, Institute of Atmospheric Physics, Chinese Academy of Sciences, Beijing, China; 10 Cold and Arid Regions Environmental and Engineering Research Institute, Chinese Academy of Sciences, Lanzhou, Gansu, China; 11 School of Geography, Beijing Normal University, Beijing, China; 12 Chinese Academy of Meteorological Sciences, Beijing, China; 13 Center for Research in Isotopes and Environmental Dynamics, University of Tsukuba, Tsukuba, Ibaraki, Japan; 14 Department of Agro-Meteorology, National Institute for Agro-Environmental Sciences, Tsukuba, Ibaraki, Japan; 15 Institute of Meteorology and Hydrology, Juulchin, Ulaanbaatar, Mongolia; 16 Research Faculty of Agriculture, Hokkaido University, Sapporo, Hokkaido, Japan; 17 Key Laboratory of Ecosystem Network Observation and Modeling, Institute of Geographic Sciences and Natural Resources Research, Chinese Academy of Sciences, Beijing, China; 18 Center for Environmental Biology and Ecosystem Studies, National Institute for Environmental Studies, Tsukuba, Ibaraki, Japan; NASA Jet Propulsion Laboratory, United States of America

## Abstract

The regression tree method is used to upscale evapotranspiration (ET) measurements at eddy-covariance (EC) towers to the grassland ecosystems over the Dryland East Asia (DEA). The regression tree model was driven by satellite and meteorology datasets, and explained 82% and 76% of the variations of ET observations in the calibration and validation datasets, respectively. The annual ET estimates ranged from 222.6 to 269.1 mm yr^−1^ over the DEA region with an average of 245.8 mm yr^−1^ from 1982 through 2009. Ecosystem ET showed decreased trends over 61% of the DEA region during this period, especially in most regions of Mongolia and eastern Inner Mongolia due to decreased precipitation. The increased ET occurred primarily in the western and southern DEA region. Over the entire study area, water balance (the difference between precipitation and ecosystem ET) decreased substantially during the summer and growing season. Precipitation reduction was an important cause for the severe water deficits. The drying trend occurring in the grassland ecosystems of the DEA region can exert profound impacts on a variety of terrestrial ecosystem processes and functions.

## Introduction

Hydrological and ecological processes are tightly coupled in arid and semi-arid regions. Over the last few decades, hydrological processes over the Dryland East Asia (DEA) regions have shown substantial changes, including precipitation, river discharge, soil moisture content, and associated changes in lakes area [1]–[4]. For example, precipitation in Mongolia had an average of 7.5% decrease in summer over the past half century [5]. A growing number of evidences indicate that the changes in the regional water cycle are altering ecosystem processes and functions in this region. Occurrences of widespread and persistent drought have increased across northern Mongolia recently, resulting in a general decrease in vegetation productivity for grassland ecosystems [6]–[8]. However, there are still large uncertainties on the trend and magnitude of available water for ecosystems in the DEA. Moreover, it is necessary to understand the changes in the spatial and temporal distribution of major hydrological variables and their dominant driving variables.

Water stress in many areas of the DEA has reached a dangerous level that strongly limited the sustainability of the region under the changing climate [9]. Previous studies showed DEA gradually shifted to dryer conditions since the middle of last century [10]. In order to accurately assess the hydrological cycle over the DEA, we need to understand the long-term water budget components such as precipitation, ET, and water balance (e.g., the difference between precipitation and ecosystem ET). ET plays an important role in linking water, energy, and carbon cycles, especially over the arid and semi-arid regions where ET dominates the water cycle and represents over 80–90% of precipitation with large heterogeneity spatially and temporally [4]. Such an accurate ET estimation will provide critical information to evaluate available water resource and improve water resource management in the grassland ecosystem of the DEA. However, the process-based ET models showed low performance in the grassland ecosystems of DEA region [11]–[12] and other similar global dryland ecosystems [13]–[16]. This is mainly because the process-based ET models are limited by their requirements for extensive parameterizations of highly variable factors [16]–[17].

Several advanced statistical methods (e.g., neural network, support vector machine, regression tree) have recently been applied to upscale the eddy-covariance (EC) flux tower measurements from local to continental and, to some extent, to global scales using remote sensing, meteorological reanalysis, and land cover data [18]–[20]. Satellite data, in particular, provides relatively frequent and spatially contiguous monitoring of surface biophysical variables affecting ET. Regression tree (RT) models can account for a nonlinear relationship between predictive and target variables and allow both continuous and discrete variables [19]. Previous studies showed that regression tree methods are not only more effective than a simple regression method (e.g., multivariate linear regression) but also easier to understand than other approaches, such as neural networks [21].

Here, we used a regression tree approach driven by long-term satellite and meteorology datasets to upscale eddy flux ET to the grassland ecosystems of the DEA region. We then applied these results with available precipitation records to assess the terrestrial water balance and the changes in the grassland ecosystems across the DEA region. The primary objectives of this paper are to: (1) derive and evaluate the capacity of a regression tree model to predict ET at a regional scale, (2) assess the interannual variability and spatial changes in ET, precipitation, and water balance (W) over the grassland area of the DEA region during 1982–2009, and (3) identify the regulative drivers for the regional water balance.

## Methods and Data

### 1. Ethics Statement

Evapotranspiration and associated biophysical variables used in this study were derived from the eddy-covariance (EC) tower sites within the DEA, with permits of the EC towers' Principal Investigators. The EC technique is a nondestructive micrometeorological observation approach. The field study did not involve endangered or protected species.

### 2. Study area

**Figure 1:**
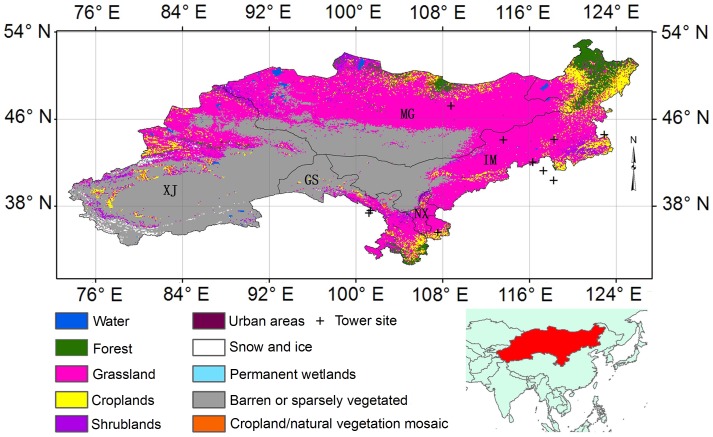
The map of the study region with land cover type and location of eddy-covariance towers. Land cover map was derived from the MODIS Land Cover product (MOD12Q1).

DEA locates in an arid and semi-arid region and includes Mongolia (MG) and four provinces and autonomous regions of China–Inner Mongolia (IM), Gansu (GS), Ningxia (NX), and Xinjiang (XJ) (Figure 1) [22]. The total area of the study is approximately 4.8 million km^2^ [23], with ∼43.3% of the land as non-vegetated area. Grassland is the most dominant cover type and accounts for 46.9% of the DEA region. This study focuses on the vast grassland ecosystems of the DEA. In eastern Inner Mongolia and northern Mongolia, annual precipitation exceeds 400 mm. Southern Mongolia and western Inner Mongolia are known as the Gobi desert where annual rainfall is <100 mm [24]. Meadow steppe, typical steppe, and temperate deserts can be found as one moves from east to west where there exists a decreasing precipitation gradient. From the grassland to the Gobi desert, the mean annual temperature in the study area ranges from <−4°C in the north area to >8°C in the central Gobi desert [25].

### 3. Regression tree method

We used the RT method of the commercial software, Cubist, to upscale tower-based EC flux ET to the grassland area of the DEA region. The RT method accounts for multivariate nonlinear relationship of the dependent variable (ET) and a set of predictive variables by producing rule-based models. RT algorithms predict class membership by recursively partitioning one dataset into many homogeneous subsets according to a gain ratio criterion [26]–each homogeneous subset following a multivariate linear least squares-type regression sub–model. Finally, all the sub–models were combined into a model tree. In this study, we used Cubist to construct a predictive ET model based on the satellite-based NDVI, meteorology inputs, and direct ET measurements from the EC towers within the DEA region. Two statistical measures were used to evaluate the performance of the model: coefficient of determination (R^2^) and mean absolute error (MAE). MAE is calculated as:
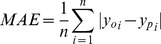
(1)where *n* is the number of samples used to establish or evaluate the model, and *y_oi_* and *y_pi_* are the observed and predicted values of the response variable, respectively.

### 4. Explanatory variables

ET is influenced by a variety biophysical variable such as soil moisture, stomatal conductance, aerodynamic conductance, incident solar, air temperature (T_a_), relative humidity (RH), vapor pressure, etc. These relationships in empirical and mechanistic methods depend also on the climate conditions [27]. For example, moisture supply in arid areas is the dominant variable affecting ET, whereas in cold regions, the temperature's influence is more pronounced [28]. Temperature and solar radiation have been widely used to express the atmospheric demand or available energy that drives ET [13], [27], [29]–[30]. Additionally, vegetation features (e.g., cover, density, or Normalized Difference Vegetation Index (NDVI) measured from satellite) are also important in quantifying plant transpiration [29] and ET [27], [29], [31]. Relative humidity and vapor pressure are important modifiers for moisture supply and limitations for ET [13], [27], [29]. Consequently, we selected NDVI, total solar radiation (R_g_), air temperature, and relative humidity as the potential drivers for predicting ET in this study.

### 5. Data from the tower sites

Direct measurements of ET and associated biophysical variables from thirteen EC tower sites in or around the DEA region were used to train and validate our RT ET model (Figure 1 and Table 1). All major climate and vegetation regions have presence of EC sites (Figure S1). The half-hourly or hourly averaged temperature, relative humidity, total solar radiation, and latent heat (LE) were aggregated into daily values (see [29], [32] for detailed procedures for processing the EC data). Because the EC flux datasets used in this study are available only after 2002 for each EC tower, Moderate Resolution Imaging Spectroradiometer (MODIS) NDVI values derived from MODIS Collection 5 subset were used in this study, which was downloaded directly from the Oak Ridge National Laboratory Distributed Active Center (ORNL DAAC) website (http://www.daac.ornl.gov/MODIS/modis.html). Quality control (QC) flags (i.e., signal cloud contamination in each pixel) were installed to accept or reject a NDVI value of low-quality data. Each 16-day MODIS NDVI value was interpolated into daily value to correspond with the interval of EC data. About 70% of the EC data were randomly selected and used as training data and the rest as validation data. Altogether, there were 3540-day measurements for model training and 1516-day measurements for model validation.

**Table 1:** Site name, location, vegetation type, and available years of the EC data used for developing and validating the ET model.

Site	Latitude, longitude	Vegetation type	Available years YYrarsyears
Arou	38.04°N, 100.46°E	Grassland	2008–2009
Qingyang	35.59°N, 107.54°E	Grassland	2009
Dongsu	44.09°N, 113.57°E	Grassland	2008–2009
Tongyu	44.57°N, 122.88°E	Prairie	2008–2009
Duolun Fence	42.04°N, 116.29°E	Grassland	2009–2010
Duolun Graze	42.05°N, 116.28°E	Grassland	2009–2010
Cn-du2	42.05°N,116.284°E	Grassland	2006
Cn-ham	37.37°N,101.18°E	Grassland	2002–2004
Cn-xfs	44.13°N,118.25°E	Grassland	2004–2006
Cn-xi1	41.25°N,117.26°E	Grassland	2006
Cn-xi2	40.36°N,118.21°E	Grassland	2006
Mg-Kbu	47.21°N,108.74°E	Grassland	2003–2008
Qhb	37.6°N,101.33°E	Grassland	2003–2004

### 6. Data over the DEA region

We used monthly temperature, relative humidity, and total solar radiation of Modern-Era Retrospective Analysis for Research and Applications (MERRA) [33] as the input for our RT ET model. MERRA is a National Aeronautics and Space Administration (NASA) reanalysis for the satellite era data using the Goddard Earth Observing System Data Assimilation System Version 5 (GEOS-5). MERRA uses data from all available global surface weather observations every three hours. GEOS-5 was used to interpolate and grid the MERRA point data over a short time sequence and produces an estimate of climatic conditions for the world at 10 m above the land surface (i.e., approximating canopy height conditions) at a resolution of 0.5° latitude by 0.67° longitude. The MERRA reanalysis dataset has been validated at the global scale using surface meteorological data to evaluate the uncertainty of various meteorological factors (e.g., temperature, radiation, humidity, and energy balance) [33].

We used the Global Precipitation Climatology Project (GPCP) Version 2.2 precipitation data [34] to evaluate the changes of regional water balance from 1982 through 2009. The GPCP Version 2.2 precipitation product combines precipitation estimates from geostationary meteorological satellite infrared data, low-orbit satellite passive microwave data, and rain gauge observations and is available at a resolution of 2.5° latitude by 2.5° longitude.

We used both the above regional climate datasets and meteorological stations observations for the climate change analysis over the DEA region. The field climate data for the climatic change analysis were from the 151 meteorological stations in Inner Mongolia, Gansu, Ningxia, and Xinjiang, provided by the China Meteorological Data Sharing Service System (http://cdc.cma.gov.cn) and 17 precipitation meteorological observations in Mongolia. The meteorological variables for Inner Mongolia, Gansu, Ningxia, and Xinjiang include air temperature, precipitation, total sunshine hour (SH), and relative humidity.

To produce a continuous time series estimation of ET for our study region, we first employed a linear regression method to combine the two NDVI products (i.e., Advanced Very High Resolution Radiometer Global Inventory Modeling and Mapping Studies (AVHRR GIMMS) NDVI during 1982–2006 and MODIS NDVI during 2000–2009) series into a single, continuous record and, then, derived the long-term ET record using the integrated AVHRR-MODIS NDVI series. The AVHRR GIMMS NDVI is based on a monthly maximum value compositing (MVC) of biweekly data with a ∼0.0727 degree spatial resolution covering the period from 1982 through 2006. The MVC is a simple method that minimizes the atmospheric and cloud contamination effects in producing quality NDVI data [35]. To be consistent with the spatial resolution of the AVHRR GIMMS NDVI data, monthly 500-m MODIS NDVI data were first spatially aggregated to ∼0.0727 degrees. The following steps were to combine the two series: (1) regress monthly MODIS NDVI on corresponding AVHRR GIMMS NDVI for the overlapping period from 2000 through 2006 using simple linear regression on a pixel-by-pixel basis, and (2) use the resulting regression equations to adjust the AVHRR GIMMS NDVI time series and compute an integrated AVHRR-MODIS NDVI monthly time series from 1982 through 2006 [36]. Throughout the growing season (April-October), the integrated AVHRR GIMMS NDVI seemed consistent with MODIS NDVI. Because the AVHRR GIMMS NDVI is available at ∼0.0727 degree resolution, the inputs were resampled to this resolution using a spatial interpolation algorithm of Zhao et al. [37].

### 7. Trend analysis

Linear trend analysis was used to analyze the regional trends in the hydrological, meteorological, and vegetation variables (*y_t_*) using a linear model (*y_t_* = *bt*+*y_0_*) [6], where *t*, *b*, and *y_0_* are the time, slope, and intercept of the regression line, respectively. The statistic *b/SE(b)* has a Student's t-distribution, and the Student's t-test was used to analyze and classify trend significance into weak, moderate, and strong categories. When |*b/SE(b)*|<1.0, i.e., *b* is within one standard deviation, the trend is classified as weak; when 1.0≤|*b/SE(b)*|≤*t_0.10_* where *t_0.10_* is the 10% critical value of the Student's t-distribution, the trend is classified as moderate; when |*b/SE(b)*|≥*t_0.10_*, the trend is statistically significant and classified as strong. These categories were further stratified into six classes according to the slopes of the statistical trends: positive weak, positive moderate, positive strong, negative weak, negative moderate, and negative strong. We also calculated regional averaged time series of hydrological variables and then applied the linear trend analysis to quantify the regional trends by seasons. We defined the spring from March to May, the summer from June to August, the autumn from September to November, the winter from December to February and the growing season from April to October.

## Results

### 1. Climate change over the DEA region

**Figure 2:**
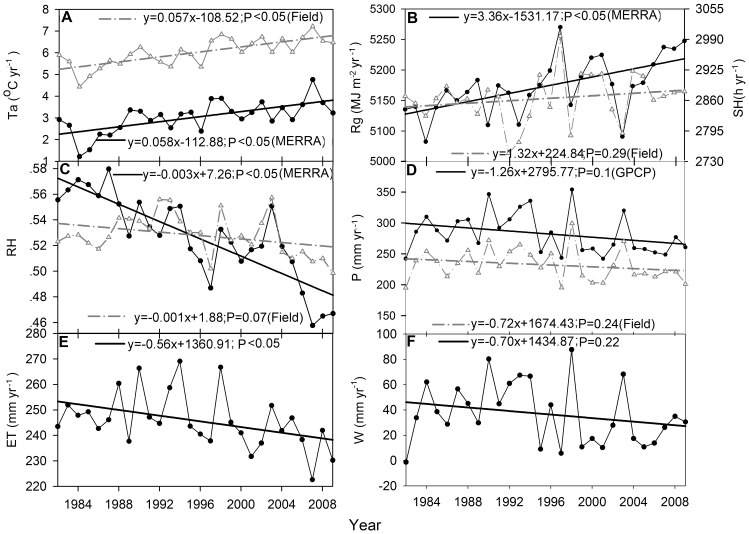
Interannual variability of meteorological variables, evapotranspiration (ET) and water balance over the DEA region. Meteorological variables includes mean annual air temperature (A), total solar radiation and sunshine hours (B), mean relative humidity (C), and total precipitation (D). The air temperature, solar radiation and relative humidity derived from MERRA are calculated over the grassland area of the DEA. The field air temperature, sunshine hours, relative humidity and precipitation are the average value at meteorological stations. The evapotranspiration is the average value of RT ET over the DEA region (E). The precipitation derives from GPCP dataset (D). The water balance is the difference between GPCP precipitation and RT ET (F).

**Figure 3:**
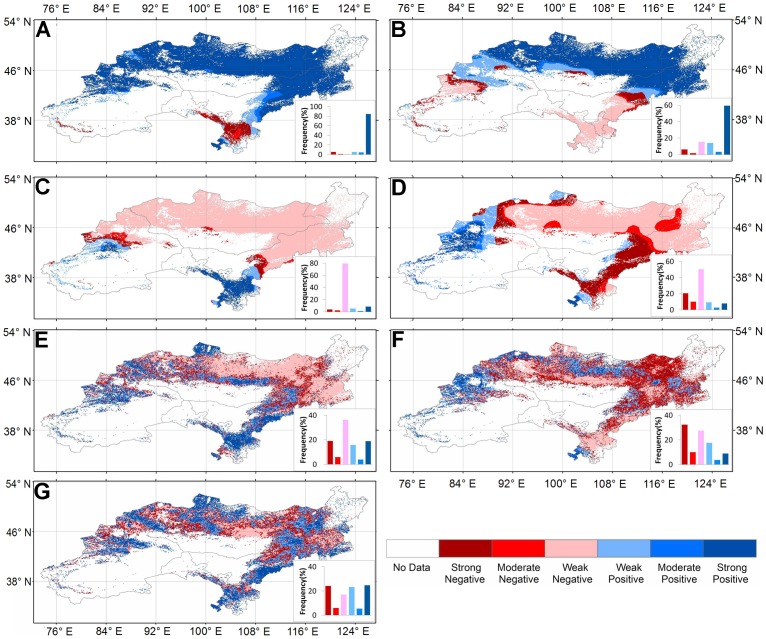
Spatial pattern of trends in meteorological variables, evapotranspiration, water balance and NDVI over the DEA region. (A)–(C) show spatial pattern of trends in mean annual air temperature, solar radiation, and average relative humidity from MERRA datasets, respectively. (D) shows the precipitation trends of GPCP dataset. (E) and (F) show the spatial pattern of trends in mean annual evapotranspiration and water balance respectively. (G) shows the trends in NDVI of growing season. The insets show the frequency distributions of corresponding trends.

From 1982 through 2009, the DEA region experienced a significant warming trend with rising mean annual air temperature of 0.058°C yr^−1^ (p<0.01) based on MERRA dataset and 0.057°C yr^−1^ (p<0.01) based on meteorological stations data (Figure 2A). Overall, temperature in about 94% of grassland areas over the DEA region increased during 1982–2009 (Figure 3A).

Solar radiation of MERRA showed a positive trend of 3.36 MJ m^−2^ yr^−1^ (p<0.01), which is consistent with that of observed sunshine hours (Figure 2B). Mongolia, northern Xinjiang, and northeast of Inner Mongolia experienced a positive trend of total solar radiation during 1982–2009, which accounts for 77% of the grassland area over the DEA region. However, Gansu, Ningxia, southwest of Inner Mongolia, and the Tianshan Mountains in Xinjiang experienced a weak negative trend (Figure 3B).

Relative humidity had a negative trend of −3.4×10^−3^ (p<0.001) based on MERRA dataset and −6.67×10^−4^ (p<0.1) based on meteorology observations (Figure 2C). Mongolia, the majority of Inner Mongolia, and northern Xinjiang experienced a weak negative trend of relative humidity during 1982–2009 (Figure 3C), which accounts for 86% of the grassland area over the DEA region. However, the positive trends occurred at Gansu, Ningxia, and the Tianshan Mountains in Xinjiang.

Precipitation had a negative trend of −1.26 mm yr^−1^ (p = 0.1) based on GPCP dataset and −0.72 mm yr^−1^ (p = 0.24) based on meteorology observations (Figure 2D). Majority of Mongolia and eastern Inner Mongolia experienced a weak negative trend of precipitation during 1982–2009 (Figure 3D), which accounts for 50% of the grassland area over the DEA region. The strong positive trend of precipitation occurred at the Tianshan Mountains in Xinjiang and southern Gansu. The strong negative trend of precipitation was found at southern DEA.

### 2. Spatial-temporal changes of ET and water balance

**Figure 4:**
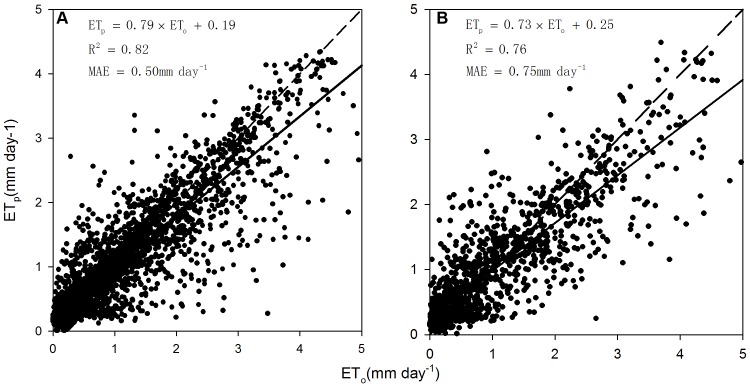
Observed (ET_o_) and predicted ET (ET_p_) with the model calibration (A) and validation (B) datasets. The short dashed lines are 1∶1 lines and the solid lines are linear regression lines.

Predicted ET values from the regression tree model agreed well with observations at thirteen EC towers, with R^2^ values of 0.76–0.82, the mean absolute error of model of 0.50–0.76 mm day^−1^ at calibration and validation sites (Figure 4). The model also successfully predicted the seasonality and interannual variations of the observed ET at the EC datasets (Figure S2).

**Figure 5:**
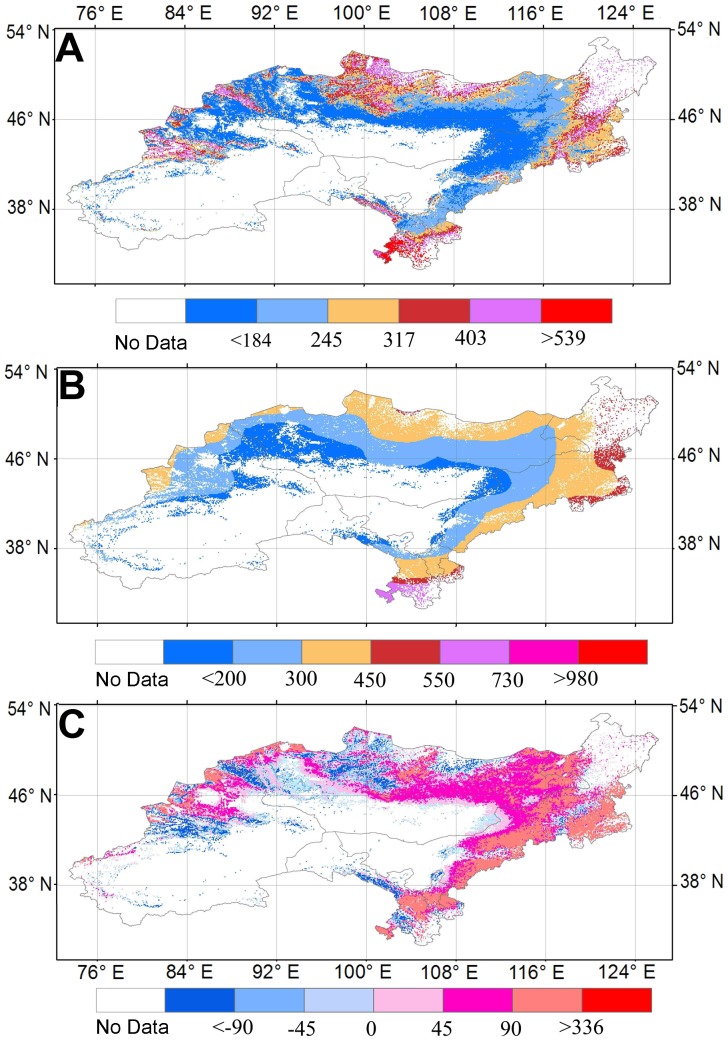
Maps of multi-year (1982–2009) mean annual evapotranspiration (ET), precipitation, and water balance. (A)–(C) show the regression tree ET, GPCP precipitation and water balance derived from GPCP precipitation, respectively.

The spatial changes of multi-year mean ET, GPCP precipitation and water balance (GPCP precipitation minus ET) from 1982 through 2009 showed an increasing trend from the center to the edge of the DEA as the land cover types changing from the Gobi desert to the grassland (Figure 5). The negative GPCP water balance was found in north-central Mongolia and the mountain areas of Xinjiang and Gansu (Figure 5C). The estimated grassland ecosystem ET ranged from 222.6 to 269.1 mm yr^−1^ with an average of 245.8 mm yr^−1^ during 1982–2009 (Figure 2E). The GPCP precipitation changed from 242 to 353.9 mm yr^−1^ with an average of 282.5 mm yr^−1^ (Figure 2D). As a whole, the mean annual GPCP water balance over the grassland area of the DEA was 36.7 mm yr^−1^, ranging from −1.3 to 87.1 mm yr^−1^ (Figure 2F).

Both ET and water balance showed high interannual variability (Figures 2E and 2F). There existed a decreased trend in annual water balance for the 1982–2009 period, mainly regulated by GPCP (−0.70 mm yr^−1^; p = 0.22) precipitation (Figure 2F), while ET showed a significant decreased trend of −0.56 mm yr^−1^ (p<0.05) over the grassland area in the DEA region (Figure 2E).

There was also considerable spatial variability in ET and water balance trends in the DEA region. The majority of Mongolia and eastern Inner Mongolia showed a negative trend in ET (Figure 3E), which was similar to the GPCP precipitation (Figure 3D) and GPCP water balance (Figure 3F), while a positive trend was observed in northern Mongolia, southern and northwestern DEA. However, the water balance derived from GPCP precipitation showed a decreased trend over the southern DEA.

**Figure 6:**
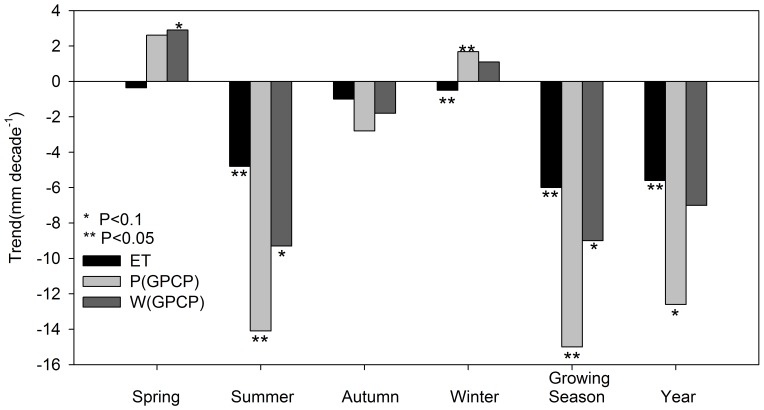
Seasonal, growing season, and annual trends of regional average evapotranspiration (ET), GPCP precipitation (P(GPCP)), and water balance derived from GPCP precipitation (W(GPCP)).

Regional average precipitation, ET, and water balance showed clear seasonal changes during the 28-year study period (Figure 6). ET had significant negative trends in the summer, winter, growing season, and annual periods, with the largest decreased trend in the growing season (−6 mm decade^−1^). The GPCP precipitation dataset showed large and significantly negative trends in the summer (−14 mm decade^−1^), growing season (−15 mm decade^−1^) and annual periods (−12.6 mm decade^−1^). The GPCP water balance also showed significant decreased trends in the summer (−9.3 mm decade^−1^) and growing season (−9 mm decade^−1^).

### 3. Correlations between ET and environmental variables

**Figure 7:**
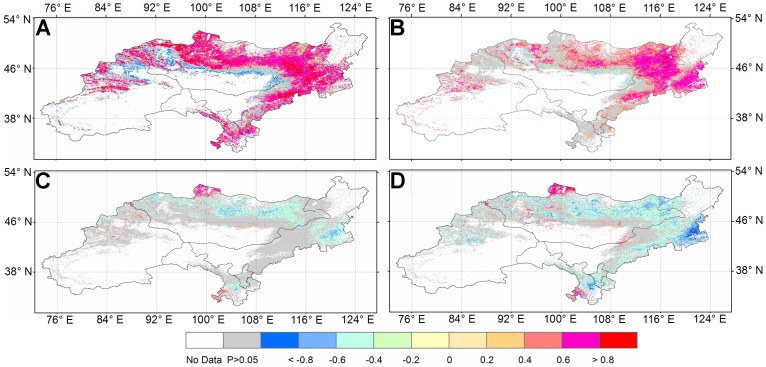
The correlation coefficients between mean annual evapotranspiration and NDVI of growing season (A), mean annual GPCP precipitation (B), mean annual temperature (C), and annual solar radiation (D). The grids with insignificant correlations were marked in gray, and other colors indicate significant correlations (p<0.05).

NDVI showed strong positive correlations (p<0.05) with ET over the majority grassland area of the DEA except the region near the Gobi desert (Figure 7A). The GPCP precipitation also showed strong positive correlations with ET over the majority of Mongolia, eastern Inner Mongolia and western DEA (Figure 7B). The temperature and solar radiation had strong positive correlations with ET in northern Mongolia and southern Gansu (Figures 7C and 7D).

## Discussions

### 1. Climate change over the DEA

The DEA region has experienced a significant and severe climate change. A few studies have reported substantial increases in air temperature in the region [38]–[39]. A strong warming of the DEA over the past three decades is firmly supported by continuous measurements from 151 meteorological stations. We found that the air temperature has increased ∼0.58°C decade^−1^ during the past three decades, which is 7.84 times to the global average (0.074°C decade^−1^) reported by the IPCC [40].

Precipitation in the DEA exhibited a slight decreased trend since 1982 with large spatial variation across the region. The precipitation of Mongolia and eastern Inner Mongolia showed decreased trend, and Xinjiang and southern Gansu provinces experienced more precipitation. Previous studies have highlighted the regional trends of precipitation over the study area [2]. However, the increased precipitation mostly occurred at desert and barren land over western DEA, and the grassland ecosystems of the DEA have experienced a drier and warmer climate change.

### 2. Model performance analysis

**Figure 8:**
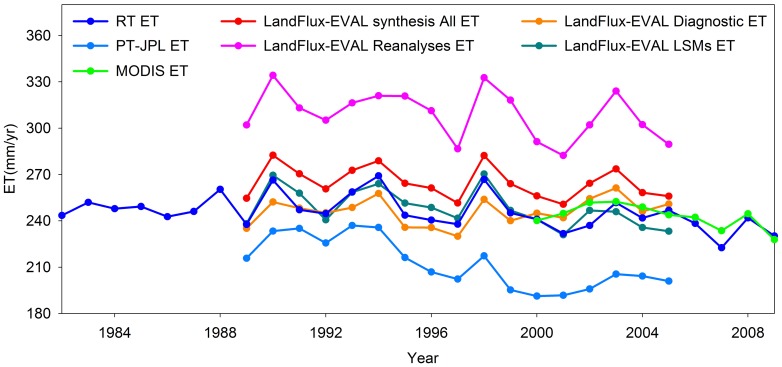
Interannual variability of RT ET, PT-JPL ET, MODIS ET and LandFlux-EVAL synthesis ET datasets. The LandFlux-EVAL synthesis ET datasets included four ensemble products from diagnostic ET data sets (LandFlux-EVAL Diagnostic ET), land surface models (LSMs) ET (LandFlux-EVAL LSMs ET), ET reanalyses dataset (LandFlux-EVAL Reanalyses ET)) and all the three categories (LandFlux-EVAL synthesis All ET).

Model calibration and validation at thirteen grassland EC sites suggested that the empirical RT approach has a large potential for up-scaling tower measurements to large areas. The ratio between the estimated ET and precipitation (i.e., 87%) for the DEA region is comparable with previous study [4]. Our estimate of mean annual ET was 245.8 mm year^−1^, which was comparable to other six ET estimations, including a Priestley Taylor type ET estimates (PT-JPL: Priestley Taylor-Jet Propulsion Laboratory) [31], MODIS ET product [13] and four ensemble datasets [41] (Figure 8). The four ensemble ET datasets [41] suggested that the annual ET ranges from 246 to 309 mm year^−1^. Two satellite-based models (i.e., PT-JPL ET and MODIS ET) estimated regional ET of 212.4 and 243 mm year^−1^ over the grassland area of the DEA respectively [13], [31]. Moreover, the RT ET showed consistent interannual variations with four ensemble ET datasets and PT-JPL ET from 1989 through 2005.

Large differences between predicted and observed ET, however, still existed at a few EC sites. For example, the RT model did not capture the exceptionally high ET values over the growing season for some sites (e.g., Cn-du2, Tongyu and Dongsu; Figure S2). The uncertainty in the driving datasets was one of the important causes. We made no attempt to improve the quality of the NDVI data where noises and errors are inevitable at the flux tower footprint scale. The noises or errors in the NDVI values, therefore, would have been transferred to ET predictions. Moreover, the accuracy of regional ET estimates depended highly on the meteorological dataset. For example, MERRA dataset tended to underestimate relative humidity over the DEA. On the other hand, the uncertainty of ET measurements may strongly impact the model accuracy. It has been noted that the sum of sensible heat (H) and latent heat (LE) as measured by the EC method is generally less than the available energy [42], which would result in part the model bias.

### 3. Terrestrial ET and water balance over the DEA

Regional climate change has produced substantial effects on water balance by altering terrestrial ET. ET showed negative trends over 61% of the DEA region, particularly in the majority of Mongolia and eastern Inner Mongolia (also see John et al. [43]), and positive trends in ET occurred primarily at the west and south of DEA. These estimates are also consistent with that of Jung et al. [30] who reported a decreasing trend of ET in drylands such as the DEA.

Precipitation plays a dominant role in regulating the variation of ET in arid and semi-arid regions [44]. McVicar et al. [45] classified the globe into energy-limited and water-limited areas based on ET and the DEA lies mainly within the water-limited area. The decreased precipitation over the majority of Mongolia and eastern Inner Mongolia seemed responsible for the negative change in ET. The increased temperature and solar radiation lead to the positive trend in ET at northern Mongolia. The strong positive correlations between NDVI and ET imply the importance of ET in regulating vegetation growth. Site study also found that the vegetation was one of major factors affecting the energy partitioning to latent heat flux [46].

Other lines of evidence also support our findings that the DEA region has experienced strong drying trends [47]–[49]. Studies based on climate station data showed that much of northern China has experienced droughts since the 1950s, with the most severe and prolonged droughts having occurred since 1990 [50]–[51]. For example, Zou et al. [49] calculated the Palmer Drought Severity Index (PDSI) for the period of 1951–2003 over China and found that >25% of the nation is under drought threat on an annual basis [51].

The drying trend occurring over the DEA region can exert profound impacts on a variety of terrestrial ecosystem structures and functions including the carbon and water cycle, plant distribution and phenology [52], vegetation growth. For example, Zhang et al. [6] reported drought-induced reductions in vegetation net primary productivity (NPP) over northern Mongolia. The negative trend of NDVI in Mongolia and eastern Inner Mongolia may be caused by the drying trend over there (Figure 3G). Moreover, recent studies found that soil moisture in north-central and northeastern China had significant downward trends, implying that northern China has become slightly drier in terms of soil moisture [53]. The results of this study show that the changes in ET and water balance over the DEA are spatially complex. The increasing water deficits in the DEA were confirmed and its impacts already happened. Future steps toward an accuracy evaluation of water balance and associated ecosystem consequences need better precipitation reanalysis data and driving environmental data.

## Conclusions

We developed a regression tree ET model driven by satellite and meteorology reanalysis datasets, and improved the accuracy of ET estimations in the dry DEA region compared with the current ET models. Using the ET measurements of the multiple eddy covariance, our validation showed the regression tree model provided the reliable ET estimations which were the fundamental dataset for analyzing regional water balance. Over the entire study area, ecosystem ET was found to decrease from 1982 through 2009, especially in the summer and growing season. Overall, precipitation and vegetation cover dominated ET changes while solar energy was found to have no significant effect. The water balance during the growing season and summer significantly decreased over the study area from 1982 through 2009. Overall, increasing water deficits in the DEA region are evident and pronounced during the study period.

## Supporting Information

Figure S1 **Distribution of eddy covariance (EC) tower sites over the climate and vegetation (NDVI) zones.**
(DOCX)

Figure S2 **Variations of daily predicted ET (ET_p_) and observed ET (ET_o_) at model validation sites.** The black solid lines represent the predicted ET and the open-circle dots represent observed ET.
(DOCX)
