# Supplementary material for: Satellite-Based Analysis of Evapotranspiration and Water Balance in the Grassland Ecosystems of Dryland East Asia
Source: PLoS One. 2014 May 20;9(5):e97295. doi: 10.1371/journal.pone.0097295 (PMC4028206; doi:10.1371/journal.pone.0097295)
Supplement: Figure S1 — Distribution of eddy covariance (EC) tower sites over the climate and vegetation (NDVI) zones. (DOCX) [file pone.0097295.s001.docx]

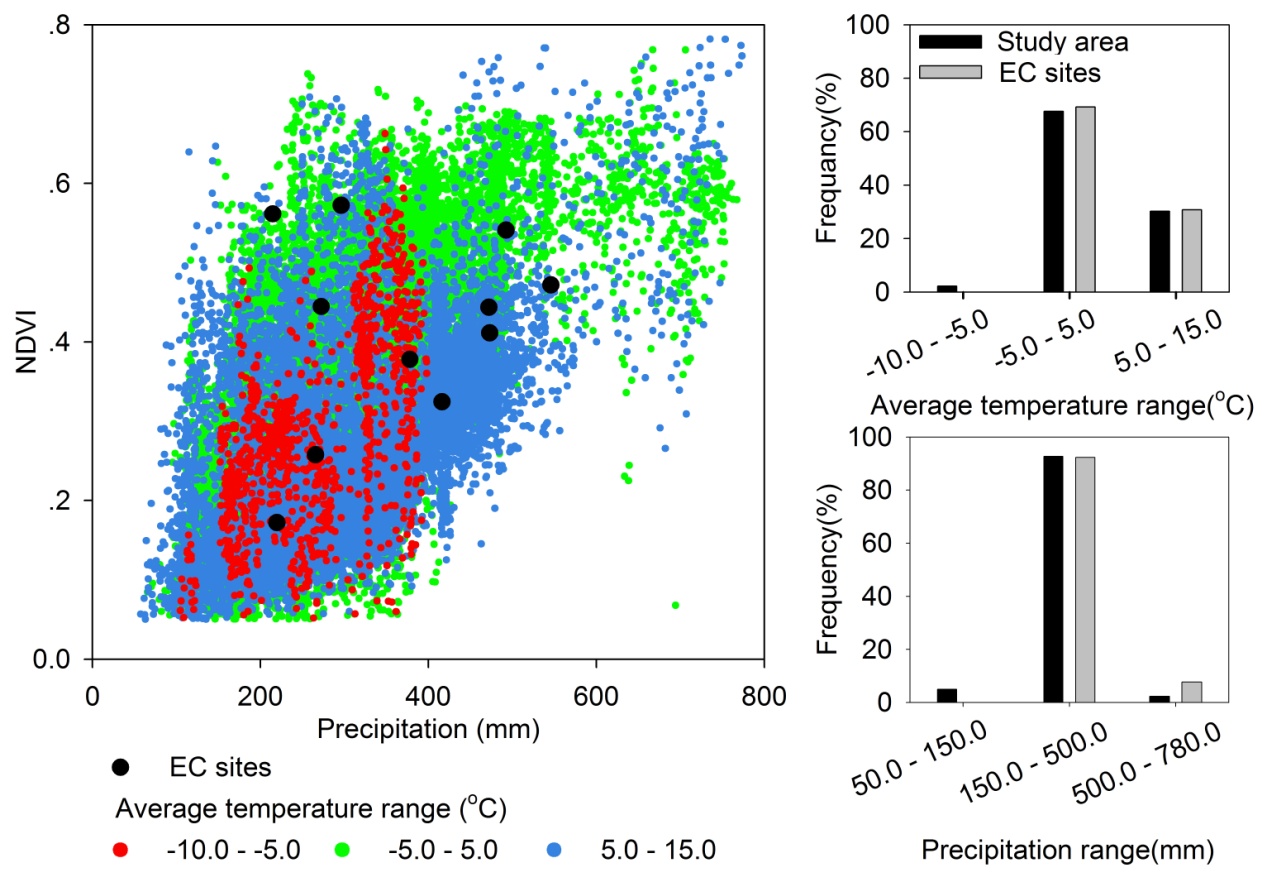


**Figure S1. Distribution of eddy covariance (EC) tower sites over the climate and vegetation (NDVI) zones.**
