# Supplementary material for: Satellite-Based Analysis of Evapotranspiration and Water Balance in the Grassland Ecosystems of Dryland East Asia
Source: PLoS One. 2014 May 20;9(5):e97295. doi: 10.1371/journal.pone.0097295 (PMC4028206; doi:10.1371/journal.pone.0097295)
Supplement: Figure S2 — Variations of daily predicted ET (ETp) and observed ET (ETo) at model validation sites. The black solid lines represent the predicted ET and the open-circle dots represent observed ET. (DOCX) [file pone.0097295.s002.docx]

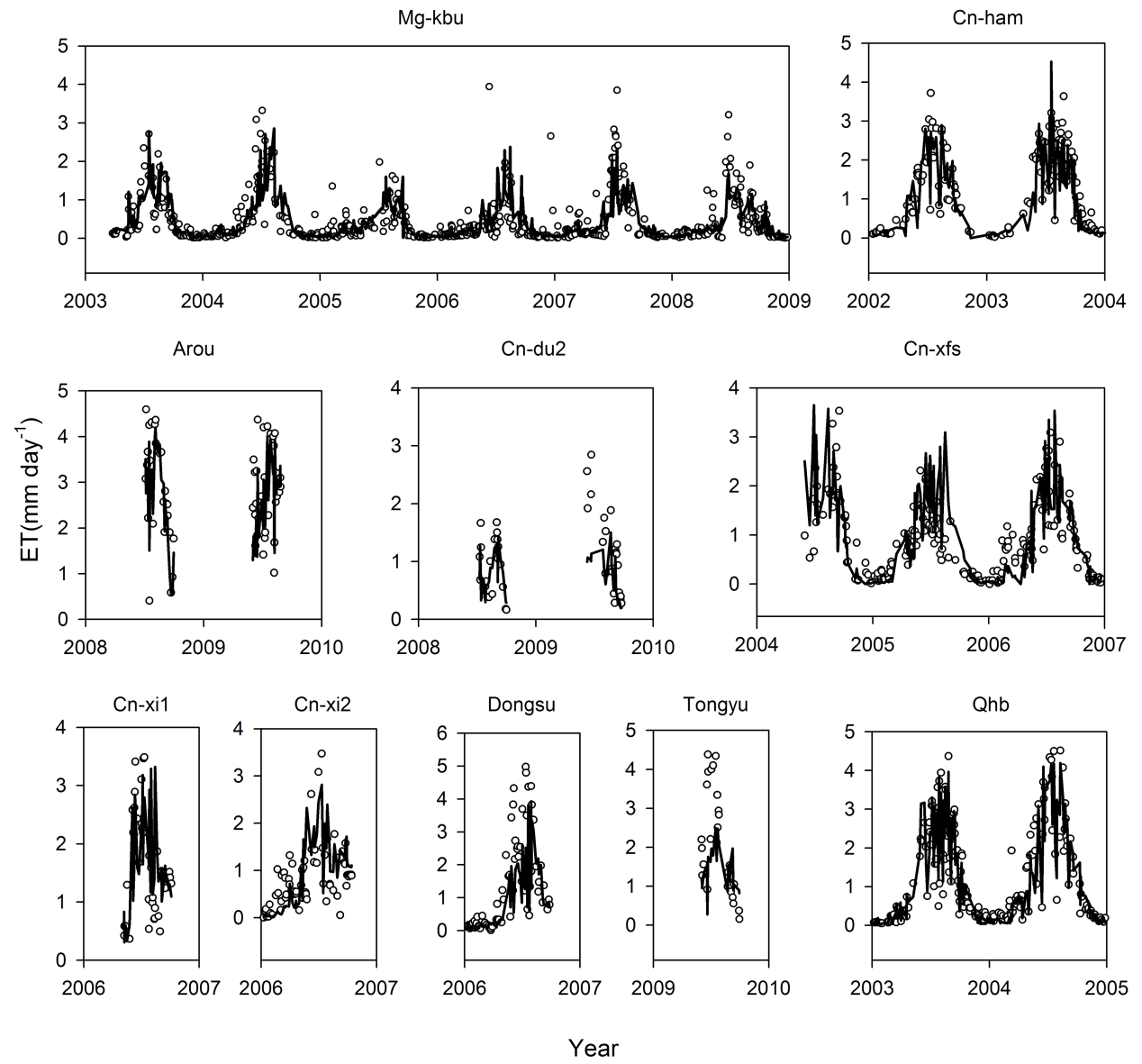


**Figure S2.** **Variations of daily predicted ET (ET_p_) and observed ET (ET_o_) at model validation sites.** The black solid lines represent the predicted ET and the open-circle dots represent observed ET.
